# Assessing the advantage of morphological changes in *Candida albicans*: a game theoretical study

**DOI:** 10.3389/fmicb.2014.00041

**Published:** 2014-02-06

**Authors:** Katarzyna M. Tyc, Clemens Kühn, Duncan Wilson, Edda Klipp

**Affiliations:** ^1^Department of Biology, Theoretical Biophysics, Humboldt-Universität zu BerlinBerlin, Germany; ^2^Department of Microbial Pathogenicity Mechanisms, Leibniz Institute for Natural Product Research and Infection Biology, Hans Knöll InstituteJena, Germany

**Keywords:** *Candida albicans*, host-pathogen interactions, pair-wise context, mathematical modeling, evolutionary game theory

## Abstract

A range of attributes determines the virulence of human pathogens. During interactions with their hosts, pathogenic microbes often undergo transitions between distinct stages, and the ability to switch between these can be directly related to the disease process. Understanding the mechanisms and dynamics of these transitions is a key factor in understanding and combating infectious diseases. The human fungal pathogen *Candida albicans* exhibits different morphotypes at different stages during the course of infection (candidiasis). For example, hyphae are considered to be the invasive form, which causes tissue damage, while yeast cells are predominant in the commensal stage. Here, we described interactions of *C. albicans* with its human host in a game theoretic model. In the game, players are fungal cells. Each fungal cell can adopt one of the two strategies: to exist as a yeast or hyphal cell. We characterized the ranges of model parameters in which the coexistence of both yeast and hyphal forms is plausible. Stability analysis of the system showed that, in theory, a reduced ability of the host to specifically recognize yeast and hyphal cells can result in bi-stability of the microbial populations' profile. Inspired by the model analysis we reasoned that the types of microbial interactions can change during invasive candidiasis. We found that *positive cooperation* among fungal cells occurs in mild infections and an enhanced tendency to invade the host is associated with *negative cooperation*. The model can easily be extended to multi-player systems with direct application to identifying individuals that enhance either *positive* or *negative cooperation*. Results of the modeling approach have potential application in developing treatment strategies.

## Introduction

The yeast *Candida albicans* is a normal inhabitant of the human microflora. It is a harmless commensal in healthy individuals, but can cause severe infections (invasive candidiasis) when the bacterial flora is removed or unbalanced (for instance, upon antibiotic treatment) or when epithelial barriers are disrupted. Additionally, immunocompromised patients are especially susceptible to fungal infections (Mavor et al., 2005). The interactions of *C. albicans* with its human host are highly complex and only partially understood. The fungus can grow in a number of different morphological forms (yeast, pseudohyphae, hyphae, chlamydospores) (Odds, 1998; Berman and Sudbery, 2002; Miller and Johnson, 2002). The two most widely studied morphologies are yeast and hyphae, which play different roles during commensal growth and infection. For example, ovoid yeast cells are the predominant form during the commensal stage or systemic dissemination (Moyes et al., 2010), while filamentous hyphae, initiated by the formation of germ tubes, are crucial for tissue invasion (Wächtler et al., 2011). A balanced epithelial microflora helps to control the *Candida* population size and possibly inhibits the formation of hyphae. An unbalanced microflora and a malfunctioning immune system are unable to control the *C. albicans* population size and the host becomes susceptible to candidiasis (Macphail et al., 2002; Perlroth et al., 2007). In these situations, the fungal burden increases and various environmental cues, such as nutrient availability, pH, and temperature stimulate hypha formation (Biswas et al., 2007; Whiteway and Bachewich, 2007; Shapiro and Cowen, 2010; Sudbery, 2011). This may lead to life-threatening infections where the fungi disseminate to different internal organs (Macphail et al., 2002; Pfaller and Diekema, 2007). Indeed, *Candida* species are now the fourth most common cause of hospital-acquired bloodstream infections (Wisplinghoff et al., 2004).

*C. albicans* yeast cells acquire nutrients provided from the host (e.g., in the gut) and the surrounding microbial flora. Hyphae can additionally invade host cells and therefore may feed directly on nutrients inside the host cells. On the host side, a critical mass of hyphae is sensed by the epithelium (Moyes et al., 2010). Infected epithelial cells secrete different cytokines, which act as chemo-attractants to stimulate activation of the innate immune system of the host. In turn, immune cells are recruited to the site of infection to fight the invading microbe. Although it is not fully understood which immune cells are crucial in preventing fungal dissemination, neutrophils (PMNs) have been shown to be successful in killing *C. albicans*, preferentially target *C. albicans* hyphae (Fradin et al., 2005; Wozniok et al., 2008; Soloviev et al., 2011) and are more effective than macrophages in killing *C. albicans* (Lehrer and Cline, 1969). These observations are supported by the fact that neutropenic patients have an increased risk of suffering from candidemia (Perlroth et al., 2007; Koh et al., 2008).

Here we have studied the impact of host neutrophil activity on the microbial population profile. We considered that within the fungal population both yeast and hyphal cells are plausible morphologies and that a cell's morphology could be influenced by the states of other cells in the population. To model the system, we used game theory principles to describe each cell as a player with two possible strategies: yeast or hypha. Such a game theoretic approach has previously been applied to describe decision-making processes in biological systems (Gore et al., 2009; Hummert et al., 2010). In the game, a player's payoff depends on the choice of the individual and the choice of the other player. A payoff becomes a measure for the successful survival and proliferation of a cell in the human microflora [similar to the definition of fitness in Renaud and de Meeus (1991) or in Hummert et al. (2010)].

The solution to the game is Nash equilibrium—a situation whereby neither player can improve payoff by changing its own strategy, whilst keeping the strategic choices of the remaining players fixed. In this work we only considered two possible changes to the players' payoffs when moving away from the Nash equilibrium. (1) Deviation from the Nash equilibrium by either player causes a decrease in payoff to both agents (the Nash equilibrium is good for both players). We will refer to a game with such Nash equilibrium as exhibiting *positive cooperation*. (2) In the other game that we considered, a change from Nash equilibrium by one player causes a decrease in its own payoff (by definition of Nash equilibrium) and an increase in the payoff of the opponent. This Nash equilibrium was attained at the expense of one player. In this study we refer to this type of equilibrium as *negative cooperation* between the players.

Using this game and the above definitions of *positive* and *negative cooperation*, we identified parameter ranges in which either morphological state has an advantage. Extension of the two-cell game to fungal populations allowed us to infer dynamics of the yeast/hypha ratio in a *C. albicans* population, along with the changes in the total population size. Although we do not explicitly model host dynamics, our model depends on parameters describing the host response, which allows us to deduce the effect of immune activity on a *C. albicans* population. We interpret the effect of host activity on the type of fungal interactions and demonstrate that both *positive* and *negative cooperation* interaction dynamics are likely to arise in fungal populations in the course of invasive candidiasis. From the perspective of the host, our model suggests that a differentiated host response toward the distinct fungal morphologies is necessary to keep the fungal population in the least pathogenic state.

## Materials and methods

### Modeling approach

We used game theory principles to study morphological transitions in a fungal population during invasion of the host. In our model, host and fungal pair-wise interactions determine the state of the fungus. Pair-wise interactions are viewed as interactions between two single *C. albicans* yeast cells, *C*_1_ and *C*_2_. While interacting, each cell can adopt two strategies: it can either remain as a yeast form (strategy y) or undergo the morphological transition, becoming a hyphal form (strategy h). The choice of the strategy will determine the cell's payoff, which we denote by *E*(*S*_1_, *S*_2_). *E*(*S*_1_, *S*_2_) describes a cell's payoff for playing strategy *S*_1_ against another cell's strategy *S*_2_. For instance, a yeast cell's payoff when playing against a hypha is denoted by *E*(*y,h*). Such pair-wise interactions are considered a 2-player game and a similar game has found application in another study on *C. albicans* survival strategies (Hummert et al., 2010). Here we describe the 2-player game in order to familiarize the reader with our choice of parameters and the role of the parameters in later analysis.

#### A 2-cell game theoretical model

Initially, we assumed that payoffs to either yeast or hyphal cells depend only on nutrition and are denoted by *E*^0^(*y*) and *E*^0^(*h*), respectively. In this case, each payoff corresponds to the maximum fitness a player can obtain in a given condition (Renaud and de Meeus, 1991). We calculated a yeast cell's payoff using an expression stemming from population dynamics (logistic growth)

(1) $$E^{0}(y)=\frac{u_{y,1}n^{\sigma }}{u_{y,2}+n^{\sigma }}.$$

**Figure 1:**
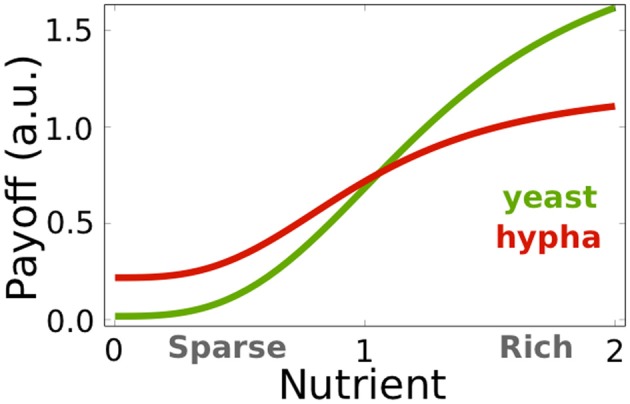
**Fungal maximal payoffs under different nutritional conditions independent of host immune activity**. Green line, *E*^0^(*y*), indicates the yeast cell's payoff in environmental conditions indicated by *n*. Red line, *E*^0^(*h*), indicates the hyphal cell's payoff under given nutritional condition *n*. As the nutrient availability decreases, the hyphae payoff exceeds yeast payoff and, hence, the hyphal state becomes advantageous. See Table 2 for the parameter values.

Here, *n* denotes nutrition provided in the microflora, *u*_*y*, 1_, *u*_*y*, 2_, and σ are morphology-dependent parameters. *E*^0^(*y*) can also be interpreted as the yeast's growth rate with relation to the level of nutrients available. As such, the yeast growth rate is negligible under conditions of sparse nutrition; increases to the maximal growth rate of *u*_*y*, 1_, and the inflection point of the increase is approximately the half-maximal speed at a nutrition provision of *u*_*y*, 2_. σ determines the steepness of increase in growth rate around the inflection point. As hyphal cells tend to penetrate tissue while yeast cells do not (Dalle et al., 2010), the payoff for hyphal cells is given by

(2) $$E^{0}(h)=\frac{u_{h,1}n^{\sigma }}{u_{h,2}+n^{\sigma }}+i,$$

where *i* describes the hyphal cell's ability to invade and subsequently feed on host cells. Both measures of the isolated fungus' payoff, *E*^0^(*y*) and *E*^0^(*h*), indicate which morphological state is favorable under specific nutritional conditions. We assume that in a healthy individual's microflora, yeast cells are predominant (Jacobsen et al., 2012). To ensure preferential yeast growth under rich nutritional conditions (Biswas et al., 2007), we assume that yeast's maximal growth rate is higher than hyphae (*u*_*y*, 1_ > *i* + *u*_*h*, 1_) and that yeasts require more nutrition for optimal growth (*u*_*y*, 2_ ≥ *u*_*h*, 2_). Figure 1 depicts an according parameterization. We set these parameters arbitrarily since, as long the above inequalities are conserved, the qualitative results of this study remain valid.

**Figure 2:**
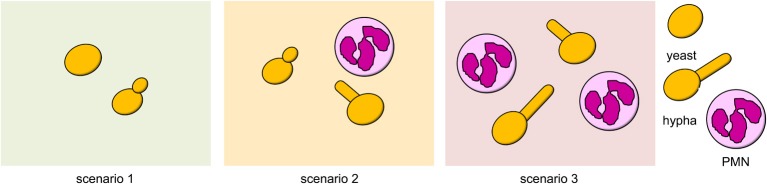
**Possible scenarios in the game**. (1) Both cells play yeast strategy. (2) Cells play distinct strategies. (3) Both cells play hyphal strategy.

Hyphae-triggered activation of the innate immune system strongly affects the success of *C. albicans* cells. Because PMNs are the key immune cells in fighting *C. albicans* infections (Fradin et al., 2005; Perlroth et al., 2007), we restricted our model to interactions between *C. albicans* and PMNs. Depending on the strategic choices of *C. albicans* cells, we distinguished three scenarios, which are depicted in Figure 2: (1) yeast cells only; (2) a mixed population of yeast and hyphal cells; (3) a pure hyphae population.

We constructed the payoff matrix for the different strategies using the following considerations:

- Since hyphae are preferentially recognized by PMNs (Wozniok et al., 2008; Jacobsen et al., 2012), the increased attraction of PMNs toward hyphal cells defines a cost for cells in the hyphal state. This cost is incorporated into the model by subtracting *k*—a parameter that indicates the efficiency of the host immune system to recognize, target, and kill hyphae—from the hyphal payoff. Thus, in a mixed scenario, i.e., when only one cell turns hyphae, the hyphae cell will score the payoff *E*(*h, y*) = *E*^0^(*h*) − *k*.
- Yeast cells are also exposed to neutrophil activity when hyphae are in their vicinity (Figure 2), but there is less attraction of neutrophils toward yeast cells (Biswas et al., 2007; Sudbery, 2011) and hence, the payoff *E*(*y, h*) = *E*^0^(*y*) − *k* · *b* with *b* ∈ [0, 1] applies. Parameter *b* explicitly describes the neutrophil's lower attraction toward yeast compared to hypha.
- We consider that each hyphal cell contributes to the activation of the immune system and hence, *E*(*h, h*) = *E*^0^(*h*) − *p* · *k* with *p* > 1 applies. Here, *p* reflects the effect of two interacting hyphal cells on the immune system activation. For simulations, we use *p* = 2.

The payoff matrix of this game is given in Table 1. The values of *p* and *b* strongly affect the outcome of this game. By requiring *b* ∈ [0, 1] and *p* > 1, we account for the neutrophils' stronger attraction toward hyphae. For *p* = 2 and *b* ∈ [0, 1) the following Nash equilibria were established:

1. All players choose “yeast” if *E*(*y, y*) > *E*(*h, y*) and *E*(*y, h*) > *E*(*h, h*);
2. Both yeast and hyphae are present if *E*(*y, y*) < *E*(*h, y*) and *E*(*y, h*) > *E*(*h, h*);
3. All players choose the strategy “hypha” if *E*(*y, y*) < *E*(*h, y*) and *E*(*y, h*) < *E*(*h, h*).

**Table 1:** **Payoff matrix for cell C_1_ (row) playing against cell C_2_ (column) in the 2-cell game**.

**2-cell game**	***Y***	***H***
*Y*	*E*^0^(*y*)	*E*^0^(*y*) *– b · k*
*H*	*E*^0^(*h*) *– k*	*E*^0^(*h*) *– p · k*

**Figure 3:**
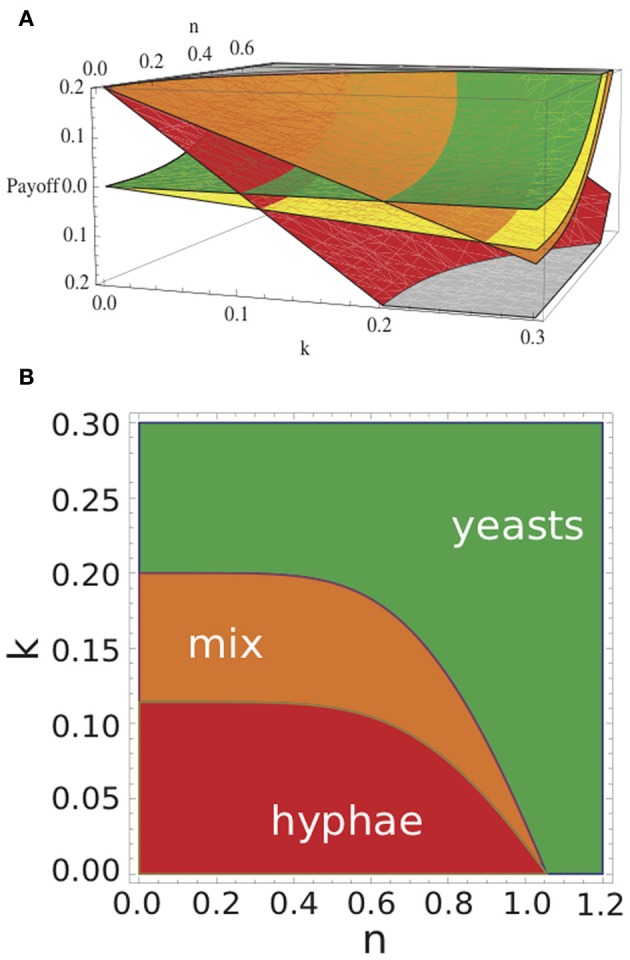
**Payoffs of *Candida* cells and Nash equilibria of the game with respect to *k* and *n*. (A)** Red plane shows the payoffs *E*(*h,h*) for hyphae in the case where both cells form hyphae. Orange plane indicates the payoffs *E*(*h,y*), i.e., for a hypha when coexisting with a yeast cell; in that case, yeast cells will gain *E*(*y,h*) (yellow plane); green plane gives *E*(*y,y*), i.e., payoff for a yeast cell coexisting with another yeast cell. **(B)** By varying immune strength *k* and nutrient *n* the outcome of the game changes. Red indicates where pure hyphae define the Nash equilibrium, green indicates where pure yeasts define the Nash equilibrium, and orange indicates parameter values where the presence of both yeast and hyphae define the Nash equilibrium. See Table 2 for model parameters.

In Figure 3A we depict the payoff values *E*(*y, y*), *E*(*y, h*), *E*(*h, y*), *E*(*h, h*) in dependence of *n* and *k* for an arbitrary parameterization (see Table 1). For example, for *k* = 0.15 and *n* = 0 *E*(*y, y*) < *E*(*h, y*) and *E*(*y, h*) > *E*(*h, h*), and hence, the Nash equilibrium establishes when both yeast and hypha coexist. All possible Nash equilibria of the game, in relation to nutrient status (*n*) and immune activity (*k*), are presented in Figure 3B: under rich nutritional conditions and high neutrophil activity, the Nash equilibrium is where both players choose to play the yeast strategy. Under poor nutritional conditions and weak neutrophil activity, the Nash equilibrium is where both players choose the hyphal strategy. For intermediate conditions, Nash equilibrium establishes whereby one player chooses yeast and the other hypha strategy. These observations are independent of the actual parameter values and remain valid whenever *E*^0^(*y*) and *E*^0^(*h*) fulfill all the constrains listed above, and *b* ∈ [0, 1) and *p* > 1.

### Derivation of replicator equation

In order to study fungal morphogenesis on a population level, we used a replicator equation. We introduced the number of yeast cells *n*_1_ and the number of hyphal cells *n*_2_ in a *C. albicans* population. *N* = *n*_1_ + *n*_2_ gives the total population size.

Hence, $x_{1}=\frac{n_{1}}{N}$ and $x_{2}=\frac{n_{2}}{N}$ represent the fraction of yeasts and hyphae, respectively. We use **x** = (*x*_1_, *x*_2_) to express the population profile, i.e., composition of yeasts, *x*_1_, and hyphae, *x*_2_, in the population. We can describe the changes in the yeast subpopulation by the following equation:

$$\frac{dn_{1}}{dt}=n_{1}\cdot E\text{​}\left(y,x\right).$$

Here, *E*(*y*, **x**) = *x*_1_ · *E*(*y, y*) + *x*_2_ · *E*(*y, h*) is the average payoff to yeast cells given the current fraction of yeasts and hyphae in the population. Similarly, the changes of the hyphae subpopulation are described by

$$\frac{dn_{2}}{dt}=n_{2}\cdot E\text{​}\left(h,x\right),$$

where *E*(*h*, **x**) = *x*_1_ · *E*(*h, y*) + *x*_2_ · *E*(*h, h*) is the average payoff to a hypha in the population. The overall changes in the fungal population size are given by

$$\frac{dN}{dt}=\frac{dn_{1}}{dt}+\frac{dn_{2}}{dt}.$$

Since $x_{i}=\frac{n_{i}}{N}$ we calculate $\frac{dn_{i}}{dt}=x_{i}\cdot \frac{dN}{dt}+N\cdot \frac{dx_{i}}{dt}$. We obtain a general expression for dynamic changes in the yeast subpopulation by deriving $\frac{dx_{1}}{dt}$ and, after a few straightforward calculation steps, we obtain:

$$\frac{dx_{1}}{dt}=x_{1}\cdot \left(1-x_{1}\right)\cdot \left(E\text{​}\left(y,x\right)-E\text{​}\left(h,x\right)\right).$$

Since *x*_1_ + *x*_2_ = 1, we set *x*_1_ = *x* and *x*_2_ = 1 – *x*. For more details see Webb (2007). For the simulation and analysis of the models described in this work, we used Mathematica 8 (Wolfram Research, 2010).

## Results

### Pair-wise interactions in the *C. albicans* population (model A)

#### Analysis of the model

Starting from the 2-cell game we derived the replicator equation for a fungal population. In this model, each strategy is played by a certain fraction of a population. Given **x** = (*x*, 1 − *x*), a vector describing the *C. albicans* population profile, where *x* is the fraction of yeast cells and 1 – *x* the fraction of hyphal cells in the population, the replicator equation given below describes the dynamic changes of the yeasts to hyphae ratio in the population (see Materials and Methods, section Derivation of Replicator Equation, for derivation of the equation):

(3) $$\begin{array}{l} \;\frac{dx}{dt}=x\text{​}\left(1-x\right)\left[E\text{​}\left(y,x\right)-E\text{​}\left(h,x\right)\right],\text{where}\\ E\text{​}\left(y,x\right)=x\cdot E\text{​}\left(y,y\right)+\left(1-x\right)E\text{​}\left(y,h\right),\text{and}\\ E\text{​}\left(h,x\right)=x\cdot E\text{​}\left(h,y\right)+\left(1-x\right)E\text{​}\left(h,h\right) \end{array}$$

From the host perspective, the population of *C. albicans* cells can be in three different states characterized by three different ranges of the value of *x*:

- a commensal and non-invasive state whenever *x* = 1;
- a state moderately hostile to the host when both yeast and hyphal forms can be isolated from the population, i.e., 0 < *x* < 1 (mild infection);
- a hostile population that is entirely composed of hyphal cells, i.e., *x* = 0 at steady state (severe infection).

The *C. albicans* population profile will evolve toward the steady state of the system given in Equation 3, which depends on the parameterization of the model and, more precisely, on the qualitative difference between payoffs.

#### Targeting yeast cells does not payoff to the host

Since the conclusions remain valid for all parameters that maintain the inequalities 1, 2, and 3, we set the parameters arbitrarily (Table 2) and discuss the qualitative behavior of the model (Equation 3), letting the model dynamics depend on inputs *n* and *k* only. When constructing the game we reasoned that host immune cells, neutrophils, tend to target hyphae cells. But what is the host's advantage from targeting yeast cells to a lesser extent in this game? Is the differential fungal recognition a result of compromise or conflict between the host and the fungi (Renaud and de Meeus, 1991)?

**Table 2:** **Choice of parameters for simulations presented in this work**.

**Definition**	**Comment**	**Numerical value**
*y*_0_	Initial yeast cell number	*y*_0_ = 1 × 10^−1^
*h*_0_	Initial hypha cell number	*h*_0_ = 1 × 10^−2^
*n*_0_	Initial nutritional conditions	*n*_0_ = 5 × 10^−1^
*k*	Immune system strength	*k* = 1.5 × 10^−1^
*p*	Maximal immune system activation	*p* = 2 × 10^0^
*b*	Immune system's attraction toward yeast cells	*b* = 2.5 × 10^−1^
*i*	Nutrients from tissue penetration	*i* = 2 × 10^−1^
*v*_prod_	Nutrient restoration rate	*v*_prod_ = 2 × 10^−1^
δ	Death processes in the model independent of a game	δ = 1 × 10^−1^
		*u*_*y*1_ = *u*_*y*2_ = 2 × 10^0^
*u*_*y*1_, *u*_*y*2_, *u*_*h*1_, *u*_*h*2_, σ	Parameters defining nutrient uptake	*u*_*h*1_ = *u*_*h*2_ = 1 × 10^0^
		σ = 3 × 10^0^

*The numerical values listed have arbitrary units*.

**Figure 4:**
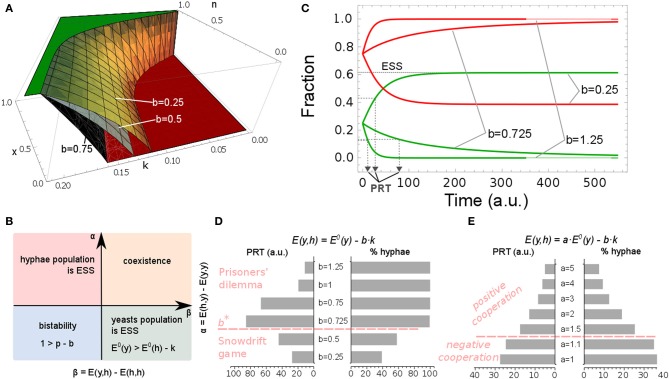
**Effect of immune system on a population profile**. Yeast fraction is given by *x*. **(A)** Population profiles for different *n* and *k*. Green plane represents pure yeast population, *x* = 1, and red plane refers to the population consisting solely of hyphal cells, *x* = 0. The switch to pure hyphae population is influenced by the values of *b*. Yellow surface for *b* = 0.25; gray surface for *b* = 0.5; black surface for *b* = 0.75. **(B)** Stability analysis of the steady states. Parameter space is divided into α and β (see Figure labels). The pure yeast or pure hyphal populations are possible evolutionary stable steady states (ESS) but coexistence of the two forms may also arise; in these cases, it is independent of the initial yeast/hyphae ratio. By altering PMNs' attraction toward yeast and hyphae forms (*d* and *b*), bi-stability in the system may occur and the final steady state is dependent on the initial fractions. **(C)** Simulation of *model A*. Parameters are set to the values given in Table 2. Population profile dynamics for different values of parameter *b* in the yeast payoff *E*(*y, h*) = *E*^0^(*y*)–*b* · *k*. For low host activity, *b* = 0.25, yeast cells are initially favored, as they increase in frequency (green line) and both cell types will coexist, as hyphal cells persist in the system (red line). Population response time, PRT, indicates the time needed for yeast cells to reach 50% of the final steady state fraction. **(D)** The immune system strength, *b · k*, influences the hyphal fraction and PRT. By increasing the yeast's cost, by setting the value of *b* to one indicated in the figure, the PRT initially increases, but after reaching a certain threshold, b^*^, the PRT decreases dramatically. The game dynamic interactions switch from Snowdrift game dynamics to Prisoners' dilemma game dynamics. **(E)** Variations in yeast maximal payoff, *E*^0^(*y*), influence the hyphae fraction and PRT. A scalar *a* has been introduced in *E*(*y, h*) = *a* · *E*^0^(*y*) − *b* · *k*. By setting the value of *a* to one, as indicated in the figure, yeast payoff is increased and PRT decreases whilst the fraction of hyphae in the population drops. At the same time, fungal interaction types change from *negative cooperation* to *positive cooperation*.

The model analysis revealed the following: an increase in the PMN targeting rate of yeast cells [i.e., by increasing the numerical value of parameter *b* in the yeast payoff *E*(*y, h*)] would result in an increase in the hyphal fraction of the population (Figure 4A) and this mixed population would become more hostile. Therefore, in our model, via parameter *k*, the host directly affects the interactions within the microbial population.

One of the classic examples in game theory is a Snowdrift game. This game models a situation where no player wants to yield to another. However, the worst possible outcome of the game is when neither player yields (Smith and Price, 1973; Hauert et al., 2006). For some ranges of parameter values, we observed increased payoffs to both players when only one of the interacting cells formed hypha and the other remained as yeast. However, the development of hyphae has associated metabolic, and other, costs, which have to be invested into hypha formation (Hummert et al., 2010). This situation would correspond to a yielding strategy described in the above Snowdrift game. Our construction of the payoff matrix (described in section A 2-Cell Game Theoretical Model) led to the conclusion that, for some ranges of values for parameter *k*, the host imposes Snowdrift game dynamics on the *C. albicans* population, enabling the coexistence of the two distinct morphologies. In this context, PMN activity can induce *positive cooperation* in the mixed microbial population, whenever

(4) $$\begin{array}{l} E\text{​}\left(y,y\right)<E\text{​}\left(y,h\right)\text{ and }E\text{​}\left(y,y\right)\text{ ​}<E\text{​}\left(h,y\right)\\ \;\left(\text{or}E\text{​}\left(h,h\right)<E\text{​}\left(h,y\right)\text{ and }E\text{​}\left(h,h\right)<E\text{​}\left(y,h\right)\right). \end{array}$$

In other words, it would require the payoffs of both yeast and hyphal strategies to be increased in the mixed scenario, compared to when both players choose the same strategy. If the inequalities in Equation (4) are satisfied, a degree of hyphal development is good for the population, as the yeast form will also benefit, i.e., yeast payoff increases. On the other hand, *negative cooperation* takes place whenever

(5) E​(y,h)<E​(y,y)<E​(h,y)(or E​(y,h)<E​(h,h)<E​(h,y)).

This means that the coexistence of yeast and hyphal cells pays off only to the hypha, whilst the yeast cell would lose in a mixed scenario.

The Prisoner's dilemma game [first formalized by Albert Tucker in 1950 (Poundstone, 1992)], another classic example from game theory, models a situation where players will choose to cheat, irrespective of the strategies of the opponent (Doebeli and Hauert, 2005). Cheating can be viewed as a way for improving a player's own payoff and at the same time worsening the opponent's payoff, i.e., our definition of *negative cooperation*. By increasing the immune response activity (expressed by *b* · *k*), the strength of *negative cooperation* in the population increases and can lead to Prisoner's dilemma dynamics. In other words, the *C. albicans* population will evolve to a pure hyphae population, even though the overall payoff would be higher if the whole population remained in the yeast morphology.

We performed a stability analysis to assess which payoff values determine different evolutionarily stable fungal populations (Figure 4B). From this, we can conclude that a stable pure hyphal population will occur whenever: (i) the difference between payoffs to isolated hyphal cells and yeast cell, *E*^0^(*h*) – *E*^0^(*y*), is larger than the host immune strength *k* (*E*^0^(*h*) − *E*^0^(*y*) > *k*), and at the same time (ii) *E*^0^(*h*) − *E*^0^(*y*) > *k* · (*p* − *b*). In contrast, a stable pure yeast population will occur whenever *E*^0^(*h*) − *E*^0^(*y*) < *k* and *E*^0^(*h*) − *E*^0^(*y*) < *k* · (*p* − *b*). We also distinguish conditions for which both yeast and hyphal forms are present in the population and when the fractions of cells playing the different strategies are stable. This will occur whenever *k* < *E*^0^(*h*) – *E*^0^(*y*) < *k* · (*p* – *b*), which implies *p* –*b* > 1. In other words, a stable mixed population will be established whenever immune activation against a population consisting of yeast and hyphal cells is weaker than immune activation against a pure hyphae population, i.e., *p* · *k* > *k* + *b · k*. Bi-stability can occur in the system whenever *k* > *E*^0^(*h*) – *E*^0^(*y*) > *k ·*(*p* – *b*), meaning *p* · *k* < *k* + *b* · *k* or equivalently *p* – *b* < 1. This implies that a system is bi-stable whenever the penalty imposed by the immune system on yeast cells, in a mixed scenario, approaches that imposed on hyphae in a pure hyphae population.

Does the host immune activity also influence the time the fungal population needs to reach a steady state? To address this question, we inspected the time needed for a population to reach half of the steady state yeast fraction. We call this the population response time, PRT (see Figure 4C, for illustration). Interestingly, small increases of the host attraction toward yeast cells, *b*, increases the PRT, so that the accumulation of hyphae in the population takes more time. However, once PMN attraction toward yeast cells passes a certain threshold, b^*^, we observed a dramatic decrease in PRT and a rapid switch to a pure hyphae population (Figure 4D). At the same time, game dynamics changed from Snowdrift to a Prisoner's dilemma game. This result suggests that the strength of the immune response determines both hyphae content and the time required to establish the stable microbial population profile. From a biological perspective, our model indicates that, in order to maintain a commensal-host relationship (predominantly yeast cells), PMN attraction to yeast cells, *b*, must remain low to prevent a rapid, population-wide transition to hyphal growth.

#### Prisoners' dilemma does not payoff for a fungal population

The payoff matrix of the game discussed here is symmetric (Table 1). Games where Nash equilibrium is given by the mixed scenario define a win-lose situation [i.e., whenever *E*(*y, h*) < *E*(*y, y*) and *E*(*h, y*) > *E*(*y, y*) and *E*(*h, y*) > *E*(*h, h*)]. Figure 4A shows simulations of such games. The bigger the distance of the Nash equilibrium payoffs in the win-lose game (by increasing the effect of immune response *b · k*), the more the hyphal form is favored. When *b · k* exceeds *E*^0^(*y*), the yeast fraction of the population becomes unsustainable and the game will result in Prisoners' dilemma where all cells will undergo the transition to hyphae, even though it would be beneficial for all to remain in the yeast form.

The higher the payoffs to cells that keep the same strategy, the lower the probability that the fungi will undergo a transition (either yeast-to-hypha or hypha-to-yeast). We introduced a scalar *a* in the payoff to the yeast cell: *E*(*y, h*) = *a* · *E*^0^(*y*) – *b* · *k*. This allows us to manipulate yeast's payoff *E*^0^(*y*) whilst keeping other parameters in the payoff matrix (Table 1) unchanged. If we increase the yeast's benefit, *E*^0^(*y*), by 1.5, 2, 3, 4, or 5 fold (Figure 4E), we generate games where *positive cooperation* is favored, since here both yeast and hyphae are winning. However, the stronger the *positive cooperation* is (through an increase in fungal payoff) the less likely the switch to the hyphal form becomes (Figure 4E).

From these simulations we can conclude that it is crucial for *C. albicans* to find a balance between yeast and hyphae benefit [*E*^0^(*y*) and *E*^0^(*h*)] to cost (*b · k* and *k*, respectively). If we relate the fraction of hyphae to the severity of infection, we can hypothesize that *positive cooperation* occurs only in mild infections. An enhanced tendency to invade the host (as expressed by enhanced hyphae formation) is always accompanied by *negative cooperation* game dynamics. It is important to note that if hyphae outcompete yeast cells, the whole *C. albicans* population will enter a Prisoners' dilemma. Furthermore, games defined by *positive cooperation* appear to result in more rapid adaptation of the population's morphological states (Figures 4D,E).

### Evolutionary dynamics in *C. albicans* population (model B)

So far we have only considered the relative changes in the yeast to hypha ratio. In the following, we drive the analysis of the system further and analyse how the different interaction types influence the overall population size. To this end, we allowed for variations in nutrient levels, which are fed into the system at a constant rate and are consumed by fungal proliferation in order to prevent unbounded exponential fungal growth. When modeling changes in the population size of interacting fungi, we describe changes in yeast and hyphal subpopulations as follows. We assume that the yeast subpopulation increases proportionally to the yeast payoff described by *E*(*y*, **x**) (see Equation 3). We also consider a possibility for *C. albicans* cells to be excluded from the game independent of the interactions with immune system, and we account for that by introducing δ. Since yeasts and hyphae have similar replicative life spans (Fu et al., 2008) we assume δ to be the same for both subpopulations. This leads to the following expression to describe changes in the yeast population size:

(6) $$\dot{y}(t)=y(t)\cdot \left(-\delta +E\left(y,x\right)\right).$$

Analogously, changes in the hyphal population size are expressed by the following formula:

(7) $$\dot{h}\left(t\right)=h\left(t\right)\cdot \left(-\delta +E\left(h,x\right)\right),$$

where the term *E*(*h*, **x**) describes the hyphal payoff.

The decrease in nutrient levels is proportional to the *C. albicans* population size. We include the influx of nutrients into the system by *v*_prod_. This gives us a formula for nutrient changes in the system:

(8) $$\dot{n}(t)=-y(t)\cdot E^{0}(y)-h(t)\cdot \left(E^{0}(h)-i\right)+v_{\text{prod}}.$$

**Figure 5:**
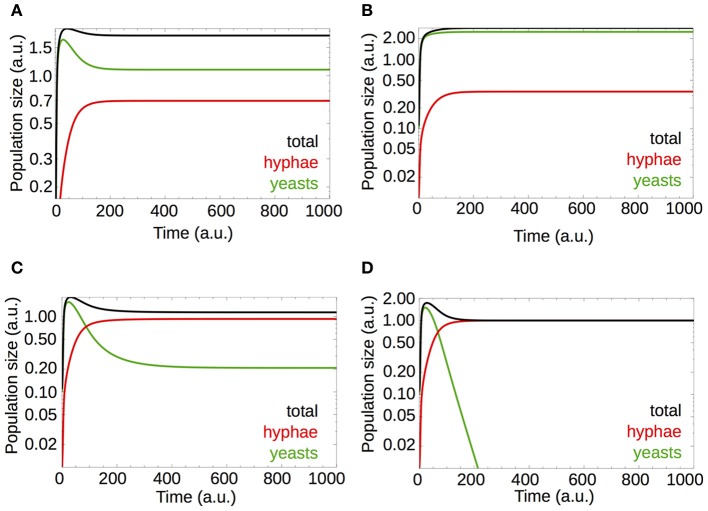
**Switch from *positive cooperation* to *negative cooperation* dynamics results in a decreased fungal burden**. Parameters are given in Table 2. **(A)** Simulations for a default parameter set, given in Table 2. **(B)** The yeast payoff *E*(*y*, *h*) = *a* · *E*^0^(*y*) − *b* · *k* modified by setting a scalar *a* to 5 (see the main text and legend of Figure 4). This increases the yeast payoff and favors game dynamic interactions triggered by *positive cooperation*. **(C)** By increasing the strength of immune system from *b* = 0.25 to *b* = 0.725, the hyphae fraction in the population is increased (see also Figure 4C), along with the total number of hyphae [compare with **(A)**] and establishes interactions with *negative cooperation* at the same time. *Negative cooperation* leads to a subtle decrease in total cell numbers [compare with **(A)** and **(B)**]. **(D)** An increase of the immune system activity from *b* = 0.25 to *b* = 1 increases *negative cooperation* between the cells and decreases the time needed for hyphae to outgrow yeast cells [compare with **(C)**].

Equations 6–8 describe *model B*. Games where *negative cooperation* takes place are necessary for establishing a hostile pure hyphae population (Figure 4D). On the other hand, if we analyse the population size (*model B*, Equation 6, Figure 5A), we observed that *positive cooperation*, obtained by setting *a* = 5 in *E*(*y, h*) = *a* · *E*^0^(*y*) – *b* · *k*, leads to a higher total population size (compare Figure 5B with Figure 5A). This result is in line with other studies, for instance, it was observed that in bacterial biofilms, the presence of a cooperative strain increases the population density (Popat et al., 2012).

Slight changes in the host immune response [by varying *b* in *E*(*y*, *h*) = *E*^0^(*y*) − *b* · *k*] lead to game dynamics dominated by *negative cooperation* and a subtle decrease in the total *C. albicans* population size (Figure 5C, *b* = 0.75). The overall *C. albicans* population decreases due to a decrease in yeast cells while a small increase in hyphae numbers is observed. Further increases of *b* lead to even more pronounced *negative cooperation* where hyphae outcompete yeasts (Figure 5D, *b* = 1).

**Figure 6:**
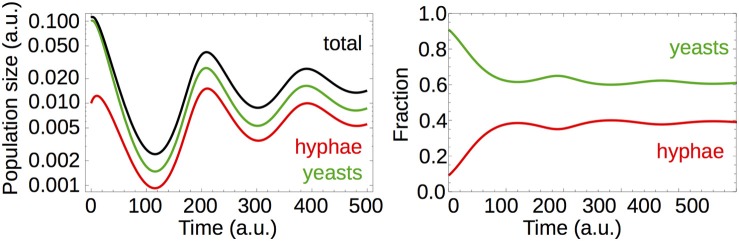
**Simulation of a population model with modified rates of nutrient restoration**. Changing the nutrient restoration rate by a factor of 0.01 induces dampened oscillations. The total population size decreases by two orders of magnitude.

In accordance with the results for the PRT from *model A* (section Targeting Yeast Cells Does Not Payoff to the Host), the time needed to reach steady populations decreases with an increase in *b* (compare Figures 5C,D). We observed that the temporal behavior of the population profile is also dependent on the nutritional conditions. Figure 6 shows that a low nutrient restoration rate may lead to dampened oscillations in the system but it also reduces the total population size.

The extended analysis of the system, where we accounted for population size, indicates that the overall *C. albicans* population is higher when *positive cooperation* takes place. Moreover, *positive cooperation* results in a lower hyphae population size. In summary, *positive cooperation* might take place in order to increase colonization levels by *C. albicans* yeast cells. *Negative cooperation* could then occur to promote accumulation of hyphae. Consequently, the hyphal state can be interpreted as a state that promotes *C. albicans* population survival in nutritionally stressful conditions or that allows the translocation of the pathogen to different habitats.

## Discussion

The work presented here is focused on the application of evolutionary game theory to study the effect of host immunity on changes in microbial population profile dynamics. Our analysis suggests that both pathogen and host benefit from the immune systems' differential attraction toward the different morphological forms of *C. albicans*. The reason for this mutual benefit is twofold:

- the host efficiently minimizes the number of invasive fungi
- the host favors the *positive cooperation* among yeast cells leading to a relative increase in yeast density.

### Possible extensions

One possible strategy for an extension of the current model would be to incorporate other existing models (Renaud and de Meeus, 1991; Gore et al., 2009; Hummert et al., 2010). Gore et al. (2009), for instance, reported experimental data supporting a model for budding cells establishing cooperative interaction strategies in order to coexist in given conditions. Hummert et al. (2010), on the other hand, presented a model of survival of *C. albicans* cells ingested by macrophages, and discussed evolutionarily stable populations related to the cost for adopting a silencing (yeast) or a piercing (hyphal) strategy. Renaud and de Meeus (1991) described a model for host-pathogen interactions where they defined strategies and payoffs for both pathogen and the host. Here, we focused on games within a heterogeneous fungal population. We focused our analysis on PMN activity as these immune cells play a dominant role in controlling fungal infections and killing *C. albicans* (Fradin et al., 2005; Perlroth et al., 2007). However, in principle, *models A* and *B* presented in the current study could be extended to other host cell types or immune components, or joined with other models (e.g., Hummert et al., 2010) to investigate the interplay of different host defense strategies. Our models largely focus on intraspecific interactions. To analyse interspecific interactions, as described for instance in Renaud and de Meeus (1991), and their impact on system's dynamics, the models may be extended to systems with more interacting cell types. In principle, one could consider a model where *C. albicans* yeast and hypha are playing against PMNs, macrophages, and/or other host cell types.

Using a game theoretic approach, we have identified situations where *C. albicans* cells engage in either *positive* or *negative cooperation*. Given experimental data on population dynamics alone (Gore et al., 2009), one can directly estimate values in the payoff matrix and extract information on what type of interactions individuals are involved in e.g., Hummert et al. (2010) and this work. Being able to infer information on microbial interaction types from mixed populations, consisting of a multitude of cell types, is of utmost importance (Tyc and Klipp, 2011). Such information could contribute to our understanding of *C. albicans* behavior at the population level, such as during biofilm maturation or the formation of lesions within host tissue. Moreover, the models presented here could be extended to describe interactions between microbial cells from different species; for example, interactions between *C. albicans* and bacteria of the commensal microbiota.

### Experimental strategy toward model validation

The main prediction made in this study is that the host immune system's lower targeting of the yeast morphology benefits the host (and the *C. albicans* commensal population). It may be possible to test this prediction by colonizing mice (White et al., 2007) with *C. albicans* wild type yeasts and yeasts over-expressing a factor which attracts PMNs. The surface-associated and secreted factor, Pra1, may be suitable for such an approach because it serves to attract PMNs to *C. albicans* (Soloviev et al., 2007; Losse et al., 2011). The fungal burden and morphology of wild type vs. *PRA1*-overexpressing cells could then be assessed to determine whether heightened PMN attraction to *C. albicans* yeast cells resulted in: (1) a reduction in overall *C. albicans* population size and (2) a switch to the hyphal morphology. Simultaneously, host factors, such as cytokines or calprotectin, a marker for inflammation (Konikoff and Denson, 2006), could be measured to determine whether the increase in targeting of yeast cells is detrimental to the host. Using such an approach, it may be possible to experimentally verify whether the host's differential targeting of fungal morphologies benefits both the fungal population and the health of the host.

### Application of game theory to host-pathogen interactions

There are many modeling strategies suitable to study host-pathogen interactions. For instance, agent-based modeling techniques are appropriate to understand the general dynamics of the interactions governed by main rules, rather than molecular details. Agent-based modeling is also useful for determining dynamics in relation to the spatial distribution of the system components (Tyc and Klipp, 2011; Tokarski et al., 2012). Our game theoretical models provide phenomenological, rather than mechanistic descriptions of the biological system. In the absence of sufficient quantitative experimental data, the qualitative characteristics of the system were studied based on relative differences rather than exact numerical values of the parameters in the model. This allowed the investigation of the different types of interactions occurring within populations in a given environment. We analyzed the biological system stemming from the characterization of the fungal strategies (2-cell game, section A 2-Cell Game Theoretical Model), through population profiles (*model A*), to predictions on the relative changes in fungal burden in different conditions (*model B*).

#### Optimization from the pathogen perspective

Here, we have considered *C. albicans* population dynamics at a single site of infection. However, similar principles may apply during systemic disseminated infections. Considering that yeast cells presumably favor dissemination to different body parts (i.e., are capable of populating distant sites) (Jacobsen et al., 2012), but that hyphae are essential for epithelial and endothelial invasion (i.e., providing access for dissemination), we propose that yeast and hyphae favor *positive cooperation* in a *C. albicans* population in order to increase the total cell numbers (fungal burden) during a systemic infection. Additionally, as yeast cells are less immuno-stimulatory, these cells may disseminate within the bloodstream without causing an extensive host immune activation, compared to hyphae. However, in the final stages of systemic infection in mouse models, development of hyphae is favored (Lionakis et al., 2011).

#### Optimization from the host perspective

Systemic candidiasis can begin via the traversal of host barriers and entry into the vascular system, followed by escape to infect internal organs. The host immune system is activated in order to prevent this and, according to the model presented here, it also monitors and controls hyphae levels by adjusting the effect of immune responses against each cell type in the microbial population. PMNs tune their response, allowing for a certain population size and tolerating yeasts, so that the hyphae fraction (and invasion and damage) can be effectively controlled. Hence, the host would not gain an advantage from killing yeast cells more efficiently and keeping the total population size smaller. If the host cells were to target yeast cells more aggressively, hyphae would accumulate and the overall probability of infection would increase.

### Global perspective

In summary, our model proposes the following interactions between the two *C. albicans* morphologies and the PMNs of the host immune system:

1. The PMN attraction rate toward different *C. albicans* cell types (Soloviev et al., 2007, 2011; Wozniok et al., 2008) can have a direct effect on the morphological make-up of the fungal population.
2. A low PMN attraction toward yeast cells allows for the coexistence of the two morphologies, but favors the yeast morphology, and thus favors both the *C. albicans* (commensal) population and the health of the host.
3. A higher PMN attraction rate toward yeast cells would cause the population to evolve to pure hyphae, which would be damaging to the host.
4. During dissemination, both morphologies are required to increase the total population size.

There remain several outstanding questions. For instance, does the PMNs' attraction toward fungal cells change at different stages of infection or anatomical niches? Do PMNs lose their hypha-over-yeast specificity as the infection proceeds? Our modeling results imply that this may be the case if yeast cells cause damage inside these niches. However, host cell damage by *C. albicans* yeast cells has, as yet, not been observed. In mouse models of systemic candidiasis it has been observed that hyphal cells are predominant in the kidney (Lionakis et al., 2011). Indeed, our model suggests an increased cost of cooperation in internal organs. It is likely that, once reaching normally sterile organs, such as the kidney, both morphological forms will be targeted by the immune system; therefore, yeast cells may lose their immune-evasion advantage here. Moreover, there are likely fundamental differences in the nature of nutrient availability. Unlike mucosal surfaces, where nutrients may be readily supplied extracellularly, host cell invasion may be essential for optimal nutrient acquisition within the kidney. Therefore, according to our model, the hyphal property of cellular invasion increases their advantage within the kidney.

### Conflict of interest statement

The authors declare that the research was conducted in the absence of any commercial or financial relationships that could be construed as a potential conflict of interest.
